# A qualitative assessment of factors influencing implementation and sustainability of evidence-based tobacco use treatment in Vietnam health centers

**DOI:** 10.1186/s13012-020-01035-6

**Published:** 2020-09-09

**Authors:** Nancy VanDevanter, Milkie Vu, Ann Nguyen, Trang Nguyen, Hoang Van Minh, Nam Truong Nguyen, Donna R. Shelley

**Affiliations:** 1 Rory Myers College of Nursing, New York University, 433 First Avenue, New York, NY 10010 USA; 2 Department of Behavioral Sciences and Health Education, Rollins School of Public Health, Emory University, 1518 Clifton Road NE, Atlanta, GA 30322 USA; 3 Department of Population Health, NYU Langone Health, 180 Madison Ave., 17th floor, New York, NY 10016 USA; 4 Institute of Social and Medical Studies, 810 CT1A ĐN1, Ham Nghi Street, My Dinh 2 Ward, South Tu Liem District, Hanoi, Vietnam; 5 Hanoi University of Public Health, No 1A Duc Thang Street, Duc Thang Ward, North Tu Liem District, Hanoi, Vietnam; 6 Department of Public Health Policy and Management, School of Global Public Health, New York University, 715 Broadway, New York, NY 10012 USA

**Keywords:** Tobacco cessation, Vietnam, Implementation, Sustainability, CFIR

## Abstract

**Background:**

Effective strategies are needed to increase implementation and sustainability of evidence-based tobacco dependence treatment (TDT) in public health systems in low- and middle-income countries (LMICs). Our two-arm cluster randomized controlled trial (VQuit) found that a multicomponent implementation strategy was effective in increasing provider adherence to TDT guidelines in commune health center (CHCs) in Vietnam. In this paper, we present findings from a post-implementation qualitative assessment of factors influencing effective implementation and program sustainability.

**Methods:**

We conducted semi-structured qualitative interviews (*n* = 52) with 13 CHC medical directors (i.e., physicians), 25 CHC health care providers (e.g., nurses), and 14 village health workers (VHWs) in 13 study sites. Interviews were transcribed and translated into English. Two qualitative researchers used both deductive (guided by the Consolidated Framework for Implementation Research) and inductive approaches to analysis.

**Results:**

Facilitators of effective implementing of TDT included training and point-of-service tools (e.g., desktop chart with prompts for offering brief counseling) that increased knowledge and self-efficacy, patient demand for TDT, and a referral system, available in arm 2, which reduced the provider burden by shifting more intensive cessation counseling to a trained VHW. The primary challenges to sustainability were competing priorities that are driven by the Ministry of Health and may result in fewer resources for TDT compared with other health programs. However, providers and VHWs suggested several options for adapting the intervention and implementation strategies to address challenges and increasing engagement of local government committees and other sectors to sustain gains.

**Conclusion:**

Our findings offer insights into how a multicomponent implementation strategy influenced changes in the delivery of evidence-based TDT. In addition, the results illustrate the dynamic interplay between barriers and facilitators for sustaining TDT at the policy and community/practice level, particularly in the context of centralized public health systems like Vietnam’s. Sustaining gains in practice improvement and clinical outcomes will require strategies that include ongoing engagement with policymakers and other stakeholders at the national and local level, and planning for adaptations and subsequent resource allocations in order to meet the World Health Organization’s goals promoting access to effective treatment for all tobacco users.

**Trial registration:**

NCT02564653, registered September 2015

Contributions to the literature

- There is little research to guide policymakers in LMICs on how to effectively implement evidence-based tobacco dependence treatment (TDT) or how to plan for sustaining improvements in clinical care.
- Our study supports the effectiveness of a strategy for implementing TDT in the Vietnam public health system and fills gaps in knowledge about barriers and facilitators to sustaining gains in treatment delivery in a LMIC.
- We also demonstrated the importance of understanding the dynamic interplay between the policy and clinical care context to in countries like Vietnam with a centralized public health system that drives priority setting and resource allocation.
- Our study findings inform context-specific strategies for increasing the translation of the WHO’s guidelines for TDT into public health practice globally.

## Background

Low- and middle-income countries (LMICs) experience 80% of the global burden of tobacco-related illness and deaths, largely due to persistent high smoking rates [1]. For example, in Vietnam, about 45% of men are active tobacco users, one of the highest rates of tobacco use in the world [2]. Article 14 of the World Health Organization’s (WHO) Framework Convention on Tobacco Control (FCTC) states that “each country shall take effective measures to promote cessation and adequate treatment for tobacco dependence” [3].

Vietnam has implemented a national smokers’ Quitline; however, like many LMICs, evidence-based tobacco dependence treatment (TDT) is still not widely available through the public health care system [4–6]. Guidelines for TDT include asking all patients about tobacco use, advising smokers to quit, assessing readiness to quit, and providing cessation assistance (i.e., the 4 A's) [3, 7, 8]. The literature also demonstrates that multisession behavioral counseling is associated with higher abstinence rates compared with written material and/or brief advice [7, 8].

There are effective strategies for implementing guideline-recommended TDT in health care settings [9–11]. These include training and coaching, embedding reminders/alerts in chart systems to prompt providers to screen for tobacco use, and creating systems for task sharing that may include referrals to national Quitlines which allow providers to delegate more in-depth counseling [10–12]. However, most studies demonstrating the effectiveness of these strategies were conducted in high-income countries (HICs). We therefore lack data on effective strategies for optimizing implementation and sustainability of TDT in low-resource settings.

To address this gap, we conducted a cluster randomized controlled trial (RCT), referred to as VQuit, that compared the effectiveness of two multicomponent implementation strategies to increase adherence to TDT guidelines (i.e., the evidence-based intervention) in commune health center (CHCs) in Vietnam [13]. Arm 1 included provider training and a tool kit with patient educational brochures and provider materials (i.e., a poster that outlined the 4 A's and desktop decision support) to remind and support providers to deliver the 4 A's. Arm 2 included arm 1 components plus a system for providers to refer patients to a trained VHW to receive three sessions of in-person cessation counseling. The referral system included a form that was completed by providers and picked up by VHWs during their weekly visits to the CHCs [13, 14] The primary outcome was provider adherence to TDT guidelines, defined as delivery of the 4 A's, and rates of referral to the VHW in arm 2.

Prior to launching the RCT, we conducted site observations, provider surveys, and qualitative interviews with health care providers who work in the participating CHCs and focus groups with the VHWs with whom they collaborate [15–17]. The goal was to inform adaptations of the planned implementation strategies, shown to be effective in HICs, to local context and culture in order to optimize implementation effectiveness in this LMIC setting [18, 19]. The pre-implementation interviews were guided by the Consolidated Framework for Implementation Research (CFIR), a widely recognized determinants framework that includes a comprehensive and multilevel set of domains and constructs that help to explain and predict implementation [20–22]. These include intervention characteristics (e.g., complexity), outer setting (e.g., policies), inner setting (e.g., practice characteristics), characteristics of individuals (e.g., knowledge, attitudes)*,* and the implementation process*.*

The baseline assessment found that health care providers and VHWs lacked the knowledge and confidence to provide TDT, primarily because they had not received training or education [15]. There was consensus that tobacco use was a public health priority but one that they were unable to address without the necessary knowledge, skills, and resources to meet smokers’ needs. Further, there was some concern about the perceived complexity of offering a behavioral intervention and having the time to deliver this service, particularly in the context of having a large number of competing priorities that are defined by the Ministry of Health (MOH) [15]. Based on the findings, modifications were made to the intervention training program including lengthening the initial training, adding a booster training, and increasing the emphasis on, and expansion of, skill-building activities as part of the training curriculum. Additional resources were also developed to address participants’ lack of experience and confidence (e.g., desktop chart to help guide patient counseling).

An analysis of the main outcomes for the VQuit study found that the implementation strategies resulted in a significant increase in adherence to the TDT guidelines (4 A's) in both study arms (*p* < .0001) [23]. The addition of the referral system in arm 2 study sites increased access to more intensive counseling which was associated with a significant increase in 6-month biochemically validated smoking abstinence rates compared to provider brief advice/counseling alone (10.5% arm 1 vs. 25.7% arm 2; *p* < .001) [14].

This paper presents findings from qualitative interviews with CHC providers and VHWs conducted at the end of the 1-year intervention period. The objective was to identify factors that influenced the impact of the implementation strategies on adherence to TDT guidelines and to explore potential barriers to and facilitators of sustaining improvements in the delivery of evidence-based TDT in CHCs in Vietnam. Our study findings inform context-specific strategies for increasing the translation of the WHO’s guidelines for TDT into public health practice globally.

## Methods

### Study setting

The Vietnamese health care system consists of four levels of administration (in ascending order): community, district, province, and central. In the rural areas, the primary access for patients seeking public health and preventive health care services is at the community level in CHCs. CHCs are usually staffed by a physician and three to five other health care workers (e.g., nurses or midwives) with a network of 8 to 10 VHWs. VHWs are responsible for implementing the Ministry of Health’s (MOH) national health promotion and prevention priority programs in their communities and conduct home visits to ensure that patients are adhering to treatment and prevention plans.

### Study design

This qualitative study was conducted with a purposive sample of 52 key informants recruited from 13 of the 26 CHCs participating in a 2-arm RCT that compared the effectiveness of two strategies for implementing TDT guidelines in CHCs in Thai Nguyen, a rural province north of Hanoi, Vietnam. The implementation strategies are described above and in more detail in previous publications [13, 14]. Semi-structured interviews were conducted at the end of the intervention period (i.e., 1 year). The sample included 13 CHC medical directors (physicians), 25 CHC nurses, and 14 VHWs who provided counseling for patients referred by the medical director and CHC providers in the arm 2 study sites. Written informed consent was obtained from all participants, consistent with the procedures approved by the New York University School of Medicine and Institute for Social and Medical Studies (ISMS) Institutional Review Boards. Interviewers were two trained masters-level Vietnamese research staff at ISMS, the partnering research organization in Vietnam. The interviews were approximately 1 h, conducted in Vietnamese, and audiotaped.

### Data collection and measures

The interview guides were informed by the CFIR domains: (1) intervention characteristics (e.g., relative advantages of the implementation strategies, complexity of delivering TDT, the evidence-based intervention), (2) outer setting (e.g., perceived patient need for tobacco cessation services, and policy influences on the availability of services and resources), (3) inner setting (e.g., relative priority of tobacco use treatment, compatibility with current workflow), and (4) individual characteristics of providers (e.g., knowledge and self-efficacy to offer treatment). Within each of the domains, we also probed how these factors might influence the potential for sustaining TDT at the level achieved after the study was completed. The interview guides were pre-tested with providers from CHCs that were not participating in the study in order to assess comprehensibility, flow, and meaning.

### Data analysis

Qualitative data from medical directors and health care providers was transcribed verbatim, translated, and English and Vietnamese transcripts. Dr. Nguyen reviewed the parallel documents to ensure “conceptual equivalence” before analysis began [24]. Two team members experienced in qualitative methods (NV and MV) systematically integrated deductive (i.e., applying codes associated with CFIR) and inductive analysis (i.e., remaining open to informative deviations) [25]. Coding began with independent reading of the transcripts to identify preliminary themes, relevant patterns, and generative questions, followed by focused coding to identify clustered concepts, organize ideas, identify major emergent themes, and then link them to relevant theoretical constructs in the CFIR. Throughout, coders met to review their coding, conduct team debriefing meetings, and reach consensus on code names and meanings [25]. To minimize bias, coding differences between the primary coders were resolved through discussions that included a third team member (DS), going back to the original transcripts, and consulting with the Vietnam field researchers. Once all transcripts were collaboratively analyzed, a detailed codebook was created. NV and MV then used the resulting codebook to code the transcripts. Analyses were conducted using ATLAS.ti qualitative software [26]. A random sample of 20% of the transcripts was independently coded by another member of the research team to establish inter-rater reliability (i.e., kappa equal to or greater than 0.80). Our reporting adheres to the Standard for Reporting Qualitative Research (SPQR) [27].

## Results

Findings are organized under the CFIR domains and the main constructs that emerged in analyzing barriers to and facilitators of (1) effective implementation of TDT and (2) sustaining improvements in the delivery of TDT. Throughout, the results reflect participants’ responses to questions about features of both the intervention (i.e., TDT) and the implementation strategies. Quotes are labeled by respondent (i.e., CHC provider (provider), medical director (MD), or VHW).

### Factors influencing effective implementation of TDT guidelines

#### Intervention characteristics

The main constructs that emerged under this domain included the following: (1) the relative advantage of the implementation strategies as compared to the prior lack of available resources, (2) perceptions about the complexity and time demands associated with delivering TDT, (3) evidence for the effectiveness of TDT, (4) cost of treatment (i.e., medication), and (5) design and quality of the implementation strategies.

Prior to participating in this project, the CHCs had no resources or training to deliver TDT. Therefore, the implementation strategies (e.g., training, referral system) were viewed as offering an obvious *advantage* compared with the previous lack of infrastructure and support to deliver TDT. Most participants in study sites that received the referral option (arm 2) described the system as reducing the burden and *time demands*, associated with offering more intensive counseling, an *advantage* that the comparison CHCs did not have. As one provider described: “I think if patients were only provided with the counseling from the health workers at the CHC and they were not referred to the VHWs, the counseling would not be very effective because the workload for the health workers at the CHC is heavy.” (Provider #14) A medical director similarly explained that “with the support from village health workers, the burden for health workers at the community health center will be reduced and the counseling becomes a system.” (MD #7) For VHWs, however, the requirement to deliver three in-person counseling sessions was described as challenging and increased the *complexity* of their role in offering treatment; this issue overlapped with comments about compatibility with current workload and is described in more detail below under the “Inner setting” section.

Many providers reflected on the *effectiveness* of the support they were now offering patients to help them quit and how this reinforced their ongoing efforts to further engage patients in treatment: “Patients find the program very helpful because they had tried to quit many times before, but they were not successful. Thanks to our encouragement, they have been able to quit.” (*Provider #3*) With greater experience, participants also became more aware of barriers to quitting and acknowledged that counseling may not be effective for all smokers. “We do not believe that 100% of the patients participating in this program are able to quit smoking. It is very difficult.” (*MD #3*) Many VHWs and providers believed that medication would improve outcomes: “if they are provided with medicine supporting the smoking cessation, they are more likely to be successful in smoking cessation.” (*MD #5*) Although a few patients were willing to purchase products, for most, *cost* was a barrier: “people are not able to afford the medicine.” (*VHW #5*) However, the absence of available medication was not reported as a barrier to continuing to engage patients in counseling, in part, due to the largely positive patient feedback providers were receiving.

Participants described the *design and quality* of the implementation strategies, such as the desktop decision support and brochures, as extremely useful, user-friendly, and facilitating patient interactions. One CHC provider explained that the materials were especially helpful in the early stages of implementation: “It was very useful for us because we did not remember all of the knowledge that we acquired in the training courses. So during the medical examination of our patients we could glance over the card [desktop chart] to see what we should talk with patients about and the order of steps.” (*MD #7*) Another CHC medical director explained that: “The information is concise, easy to read, and easy to understand. The materials are not as long as those in other programs. We can skim the materials quickly.” (*MD #6*) A VHW also emphasized the value of the patient educational materials in facilitating their counseling role: “It helps health workers introduce the program to patients more easily.” (*VHW #5*)

#### Individual characteristics

The implementation strategies were consistently described as increasing knowledge and self-efficacy related to treating tobacco use, the two main constructs that emerged in this domain. One provider noted that “before the training course, we had no idea about providing patients with counseling on smoking cessation. After the training course, I got more knowledge on how to advise our patients to quit smoking and each step of the smoking cessation process.” (Provider #5) Another provider described: “After the training course, I have been more self-confident because I was provided with knowledge,… it is easier for me to discuss with patients.” (*Provider #7*)

The skill-building and role-playing exercises were described as particularly helpful in building confidence: “Our communication skills with the people who want to quit using cigarette or waterpipe tobacco have been improved. I have been more confident in my counseling skills and my work for the project.” (*VHW #3*) A medical director also noted, “As I had a chance to practice role-playing, I remembered the lessons better.” (*MD #2*) Similarly, as described under the previous domain, other components of the implementation strategies (e.g., training, provider support systems) offered the reinforcement and support that increased confidence.

#### Inner setting

The four main constructs that emerged within this domain included the following: (1) tension for change, (2) the relative priority of treating tobacco use, (3) compatibility of TDT with current workflow, and (4) leadership engagement*.*

*Tension for change* was reflected in the consistent comments about the discrepancy between the high prevalence of smoking among men and the lack of organizational and individual capacity to deliver treatment. There was broad agreement among participants that building the capacity to treat tobacco use was important: “I think it is very necessary and important because many people suffer from tobacco harms.” (*VHW #8*)

However, participants were mixed in their beliefs about the *relative priority* of tobacco cessation services in comparison to the large number of public health programs that they were required to implement. Almost half stated that they place the cessation program at the same level of priority as other MOH programs: “All programs are equally implemented.” (*Provider #5*) However, about one third stated that tobacco cessation should also be elevated to a national priority program: “When we understand about tobacco harms, we realize that the prevention must be given top priority.” (*Provider #6*) Another noted, “I give higher priority to the tobacco cessation program because the program brings about immediate benefits for people, as it helps people quit smoking.” (*MD #1*)

Responses to questions about the *compatibility* of integrating TDT in current workflows were also mixed. This construct was often described in association with challenges created by *competing priorities*. The most common concern was the heavy workload in several CHCs. For example, one provider noted: “If patients come to the community health center on the vaccination day, we provide medical examination to them and let them go. We cannot stay on to provide counseling.” (*Provider #3*) Another reflected: “It is easy [to deliver TDT] when there are not many patients, but it is difficult when there are many.” (*Provider #10*) A director described having to implement “a dozen national healthcare programs and the regular medical examination work concurrently.” (*MD #6*) As described under the “Intervention characteristics” section, providers with the option to refer patients to the VHW viewed delivering treatment as less burdensome; however, they too described conflicts that arose due to other priority programs.

Despite the clear conflicts created by a range of other clinical responsibilities, about half of providers described their commitment to delivering some type of tobacco cessation support even when they did not have time to complete the full 4 A's: “Sometimes we have to skip some steps because of the limited amount of time, especially when we are on a [public health] campaign or our workload is too heavy.” (*Provider #20*) This appeared related, again, to the high priority attributed to TDT in the context of the high smoking prevalence among men in Vietnam. The providers and medical directors who were most positive about the intervention’s compatibility with current workflow described making screening and counseling part of the routine primary care visit, which reduced the perceived time burden: “As the screening and counseling on smoking cessation are integrated into our work, it takes only a few minutes more, so it is okay.” (*MD #12*)

Providers across CHCs described a high level of *leadership engagement* (i.e., commitment and involvement) in promoting consistent TDT. Many participants described their leaders’ behavior as reinforcing the importance of integrating TDT into routine care: “Beyond the time for courses, the head of the community health center has also joined us in implementing activities.” (*Provider #5*)

### Factors influencing sustainability

The study identified both facilitators and barriers to sustaining evidence-based TDT that emerged primarily within the outer setting (i.e., policy environment, incentives, and patient needs), inner setting (i.e., resources, compatibility, and goals and feedback), and intervention characteristic (i.e., adaptability) domains. Engaging the MOH was central to the feasibility of implementing the project, and their role in the stakeholder advisory committed helped shape the implementation strategies and process. However, in this analysis, participants described government policies primarily in relation to sustaining components of the implementation strategies to support TDT. Therefore, we present findings in the outer setting domain in this section only.

#### Outer setting

The main challenge that providers described to sustaining the program was related to the policy environment. There was a consistent belief that, overall, cessation was not prioritized by the MOH, exemplified by the following comment: “Compared with other health issues, the support for patients in smoking cessation is not given priority.” (*MD #5*) Providers further explained that although there were other “projects on smoking prevention and cessation, they were implemented at higher levels only, not at the community health center.” (*MD #6*) MOH policies, therefore, appeared to conflict with the relatively high priority CHCs themselves attributed to offering TDT (described under the “Inner setting” section).

The low priority given to TDT, at the CHC level, was described as hindering the flow of *incentives and* resources to CHCs needed to sustain VQuit: “In other programs, like the vaccination program, the program secretaries are provided with [a financial] allowance.” (*MD #5*) Another participant emphasized the need for parity with other programs: “If we want the program to be continued, this program should be handled the same way as the national health programs.” (*Provider #4*)

However, as cessation services became available through the VQuit program, *patient needs* were driving greater demand. A CHC provider explained: “The more people come to us for smoking cessation and the more people are successful, the more people trust us, the more people come.” (*Provider #14*) Another agreed: “It is effective in the fact that when one person comes to us, many other people also follow.” (*Provider #15*)

The increase in demand was viewed as facilitating support among local policymakers and other organizations. A CHC director described the effect of the intervention’s impact on the wider community: “The People’s Committee (a non-MOH, local government committee) has also been very interested in this issue. Different sectors and mass organizations have greatly supported this program and joined us in implementing this program.” (*MD#3*) Several medical directors reported plans to strengthen their partnerships with the commune-level People’s Committee and other local- and district-level committees to obtain support to sustain TDT as a routine part of CHC and VHW services. A medical director explained that “with the support from the commune People’s Committee, we get the involvement from all sectors and organizations, at commune, village, and hamlet levels, so everything is okay.” (*MD #8*)

#### Inner setting

Medical directors, in particular, described the need to sustain the *resources*, including training and other materials that were components of the implementation strategies made available through the VQuit project: “The program’s activities will be maintained if the project provides funding, flyers, and enhanced training courses.” (*MD #7*) Another provider agreed that “funding is an important source to maintain and implement the program.” (*Provider #8*)

Continued funding was identified as a particular concern related to sustaining the VHW-delivered multisession intervention in the context of their already heavy workload. About half of the VHWs and providers predicted that, without the study’s stipend, VHWs were unlikely to continue providing counseling at the level prescribed in the current protocol. One VHW shared that: “Compared to all other programs, the tobacco program is more time-consuming, so I want to be provided with more allowance.” (*VHW #7*) Another noted that if they integrate counseling into the usual work of the VHW, “My workload [would be] heavier. The counseling work is time-consuming.” (*VHW #5*) Despite barriers to sustainability created by time demands and losing the stipend, most VHWs described a commitment to continue this work, although again, not at the level the study required: “They [VHW] will not stop the program, but they will not spend much time on it. Resources are not sufficient to do so much work.” (*VHW #8*) Another suggested that: “We can remind them [the patients] to quit, but we will not follow the smoking cessation process as closely as while the project was being implemented.” (*VHW #2*)

Providers did describe competing demands in the context of discussions about sustainability, but the expectation to deliver only brief advice and counseling during routine patient visits was viewed as consistent with their role and aligned with their current practice. Therefore, most providers described sustaining the treatment protocol as feasible: “I think we still provide counseling and support.” (*MD #7*) As noted in the previous section, several participants felt the tobacco intervention had been so well integrated into the ongoing services of the CHC that it would continue after the study was over: “When the project ends and does not provide funding for this program anymore, we will still continue to implement the program. At that time, it has become habit. We have a foundation.” (*Provider #7*)

Another potential challenge to sustaining gains in TDT, however, was the lack of data tracking systems to monitor the impact of the intervention (*goals and feedback*). Several medical directors and VHWs *raised* this concern: “Other programs have their own targets. Targets are needed; otherwise, the program will be overwhelmed by other programs and will be forgotten.” (*MD #3*) “Without monitoring the work, the counselors don’t know if their counseling work is effective.” (*VHW #5*)

#### Intervention characteristics

VHWs suggested several ways to increase the feasibility of sustaining their role in treating tobacco use by *adapting* the intervention to reduce the burden of in-person visits. Suggestions included integrating VHW counseling into other community meetings: “For the community communication, we can provide counseling during meetings or other events.” (*VHW #7*) Providers and VHWs both suggested “integrating [TDT] into other programs like the pulmonary health program.” (*VHW #4*)

## Discussion

Our post-intervention assessment found the implementation strategies (i.e., training and changes to the practice environment and workflow) were well received and facilitated uptake of TDT among providers and VHWs. The strategies were designed to address multilevel barriers identified through a formative participatory research process during which the strategies were adapted and tailored to the public health and practice context in Vietnam [15]. This post-test analysis further expanded our understanding of both facilitators and persistent challenges to implementing and sustaining TDT across multiple CFIR domains.

For example, the arm 2 workflow changes that gave providers the option to refer patients to a VHW for more intensive counseling reduced the time required to deliver TDT and, therefore, increased the feasibility of integrating the intervention into routine care. This example of task shifting, or delegating the more intensive cessation counseling to the VHWs rather than asking the CHC health care providers to adopt this role, is a staffing model recommended by the World Health Organization to strengthen and expand the health workforce to address the demands of chronic disease prevention and treatment [28]. In the formative research, this was viewed as highly feasibly and aligned with VHWs’ role [15, 17]. However, once in the field, VHWs were more likely than CHC providers to describe challenges integrating the comparatively more complex role (i.e., 3 in-person counseling sessions) into their already broadly defined responsibilities. Challenges to implementing and sustaining evidence-based interventions clearly differ based on roles and responsibilities [29]. Therefore, redesigning care processes requires identifying current interactions and coordination among multiple team members and how each member is implementing their specific roles. During the formative research phase of the study, guided by CFIR, we examined the current workflow in the CHCs to identify potential implementation challenges and how the implementation strategies would promote and support uptake of new TDT-related care processes [15]. Workflow mapping is a collaborative process that results in a clear picture of the actions, steps, or tasks that providers currently perform and identifies opportunities for change to increase efficiency and improve care outcomes [30]. However, VHWs work outside of the clinical setting, and the specifics of their workflow were reported rather than systematically observed and documented which may have identified unanticipated challenges. Future research should incorporate prospective assessments of how variation in workflow processes, and other system characteristics that impact implementation effectiveness and sustainability, may differ across implementers and the settings where they work [29].

Competing demands created by the large number of MOH public health programs that CHCs and VHWs implement were also described as a potential barrier to sustaining TDT in Vietnam’s centralized health system where policy and program priorities are set at a national level. This leaves little room for local input and autonomy to create and sustain new programs and may explain the discrepancy between the reported supportive organizational climate for treating tobacco use in CHCs and the challenges providers anticipated in maintaining the same level of cessation service once the project ended. However, a few medical directors showed agency in independently raising the visibility of the program with community partners, specifically local government group and non-governmental organizations. Prospectively engaging and strengthening these partnerships was a strategy suggested for facilitating local financial and community support that could supplement MOH resource allocations. This finding highlighted the importance of engaging implementers to identify opportunities for partnerships that conversations with leaders at other levels of government and the health care system may not recognize.

Vietnam has made tremendous progress implementing the FCTC, and improving cessation services is receiving more attention from the Vietnam Tobacco Control Program, particularly with the recent creation of a Tobacco Fund (formerly the Vietnam Steering Committee on Smoking and Health) [31]. However, the perception among frontline providers and VHWs is that TDT is not yet specified as a priority program at the local commune level. Feedback from study participants suggested integrating TDT into existing chronic disease prevention and treatment programs rather than continuing to fund individual disease-specific programs. TDT is recommended by the WHO as part of a comprehensive package of essential services for prevention and control of non-communicable diseases (NCD) in primary care, and yet implementation of WHO guidelines for TDT continues to lag, especially in LMICs [5, 32]. More research is needed to develop effective strategies for implementing TDT as part of national NCD programs in LMIC. As political support, funding, and health system infrastructure evolve to reduce the burden of NCDs, an integrated approach may increase the potential for sustaining and scaling TDT. These strategies will vary based on political and sociocultural contexts, resources, and infrastructure, but should include investments in ongoing stakeholder engagement at the local and national level, and technical assistance to support the integration of affordable and effective primary care models for TDT into existing national public health programs that are addressing NCDs [29, 33, 34].

Finally, the study responds to calls for research to advance models for facilitating sustainability, an area of research that is particularly undeveloped in LMICs [29, 35]. In a recent research agenda setting process, authors concluded that developing and testing models of intervention sustainability was a high priority [22]. This study demonstrated the applicability of the CFIR in analyzing factors influencing sustainability in the context of the Vietnamese health care system. The CFIR was particularly useful in identifying the interplay between the inner (e.g., compatibility) and outer setting (e.g., policy environment). Study participants’ responses to questions within these domains emphasized the importance of understanding how policy priorities are determined in a given context. “Paying attention to, and fostering, interactions among influencers and implementers at multiple levels” [35] could facilitate the development of shared goals and inform necessary adaptations to sustain the implementation strategies that promoted TDT and the consistent delivery of TDT services.

There are other frameworks, however, that may provide additional explanatory value and guidance for optimizing sustainability in the sociopolitical context of countries like Vietnam [21, 36–39]. For example, Schell et al.’s public health sustainability framework emphasizes factors particularly relevant to the Vietnam context and described by participants as important determinants of sustainability [36]. These include political support and external funding stability, without which, in countries with political systems like Vietnam, implementation, sustainability, and scale-up are unlikely. In a recent analysis of responses from 127 countries that are Parties to the FCTC, a lack of political prioritization of cessation support and funding was the most commonly cited barrier to implementing Article 14 [33].

Other sustainability frameworks offer additional guidance by capturing the dynamic, iterative, adaptation process of going from dissemination to adoption, implementation, and sustainability [37, 38]. VQuit contributes to this literature by demonstrating, in a real-world context, the importance of a rigorous, iterative adaptation process at each stage of research that is responsive to feedback from policymakers and frontline providers. This type of process is more likely to generate policy- and practice-relevant data for those stakeholders responsible for implementing and sustaining TDT interventions.

Study limitations include the fact that qualitative interviews were conducted in CHCs located in rural areas of North Vietnam and may not reflect the environment in other parts of the country. However, the centralized nature of the health system makes it likely that the same challenges would be found across the country and in other health care systems with a similar social political context. In the Vietnamese cultural context, there was also a risk of a social desirability bias in the responses. However, the full range of comments, both positive and negative, suggests that we obtained candid responses.

## Conclusion

Our findings offer insights into how a multicomponent implementation strategy impacted the uptake of evidence-based TDT in the public health system in Vietnam. In addition, the results illustrate the importance of understanding the dynamic interplay between barriers and facilitators for sustaining TDT at the policy and community/practice level, particularly in the context of centralized public health systems like Vietnam’s. Sustaining gains in practice improvement and clinical outcomes will require strategies that include ongoing engagement with policymakers and other stakeholders at the national and local level, and planning for adaptations and subsequent resource allocations, in order to meet Article 14 goals.

## Data Availability

Please contact Donna Shelley, MD MPH, at donna.shelley@nyu.edu for data requests.

## Abbreviations

CFIR: Consolidated Framework for Implementation Research
CHC: Commune health center
FCTC: Framework Convention on Tobacco Control
HICs: High-income countries
LMICs: Low- and middle-income countries
MOH: Ministry of Health
TDT: Tobacco dependence treatment
VHW: Village health worker
VINACOSH: Vietnam Steering Committee on Smoking and Health
